# Supplementary material for: Acceptance and compliance with micronutrient powder (MNP) among children aged 6–23 months in northern Nigeria
Source: PLOS Glob Public Health. 2022 Oct 17;2(10):e0000961. doi: 10.1371/journal.pgph.0000961 (PMC10022258; doi:10.1371/journal.pgph.0000961)
Supplement: S1 Dataset — (ZIP) [file pgph.0000961.s008.zip › S8_Dataset/In-depth Interviews.zip!/In-depth Interviews/110715_K_DW_AB_2_CG.pdf]

INDEPTH INTERVIEW WITH FEMALE CAREGIVER USING MNP IN RAFIN GORA COMMUNITY, DANKO WASAGU LOCAL GOVERNMENT AREA OF KEBBI STATE

INTRODUCTION

I: Good morning ma, could you please describe for me the people living in your immediate household?

R: We are eleven in this household

I: I want to know how many children from the age of 6-23 months in this household?

R: I only have one male child and also a nineteen months daughter from my maid

I: If I may get you they are two

R: Yes, they are two

I: Apart from the nineteen months daughter, is there anybody again?

R: Except this one

I: And old is he?

R: I think he is about seven months

I: Thank you very much, previously when we were here we heard about some food challenges related to seasons that children do consume, could you please tell me about how you cope with them?

R: We do give them tuwo

I: And what again

R: And if we have rice, we do give them

I: I really want to know the type of food that children between 6-23 months do consume

R: We do give them beans or kunu to drink at times

I: Do you actually have any challenges feeding children between 6-23 months?

R: Actually it is the tuwo we give them and kunu together with rice and at times we give them fura

I: You give them fura with what?

R: We give them fura with milk and sugar

I: One thing I heard in other interview is that caregivers give their children "tea", I would like to know what tea means please?

R: Tea is when you add milk and bournvita together with sugar but actually we are not use in giving our children this tea except bread that can be easily be share among the children but it is difficult to make or buy tea like that for children to drink

I: Thank you very much, now I just want you to tell about your experience with the MNP that was given to you. Tell me your story about it.

R: When I started giving him, he didn't do anything and he did not fall ill. After that, when I started giving him I have not missed a single day, every day I do give him the product. When giving him the product, I have not experienced or see any side effect on him. As you can see him like this he hardly go to anybody, when am tire of carrying him I will bring him down to play with the children and if he is hungry again I will come and give him either tuwo or kunu to drink. If he finished either eating or drinking I will backed him and he will be sleeping.

I: Thank you very much, like this very MNP how do you give him?

R: They said, if I must give him the product, I should fetch kunu in a clean cup or bowl and if my hands are dirty I should wash them before giving him. So when am about to give him, I will fetch small kunu in a cup add little water then wash my hands and use one of my finger to stir the product and when he has finished taken it, I will now give him another kunu to drink, frankly speaking that is how I do give him.

I: Well done job, which type of kunu do you give him, is it a hot kunu or cold one

R: When I finished preparing the kunu, I will wait until it get cold, I will now fetch and add the MNP and give him to drink not the hot one and at the same time not that too cold.

I: Since you started giving your child this MNP could you tell the changes you have seen in your child?

R: Actually, his body has really changed. Before he was not having body like this people were saying that the boy is sick with a particular type of sickness called *bori* and I was treating it but no improvement before this MNP was brought, when I started giving him the product he started adding weight as you can see, actually the product is good

I: Is like I heard you call one sickness bori, what is bori

R: I s a medicine for gojijima

I: What is gojijima again?

R: Laughing, you mean you don't know goji, you don't have a sickness for small children called goji where you came from?

I: Yes, may be it may have a different name, describe to me the nature of that sickness

R: Yes you have, you will see a child will be stooling bit by bit, the child will emaciate do you understand

I: Yes I do, thank you very much. Please I would like for you to tell me about any barrier you faced while using the product?

R: You men the product

I: Yes the product

R: No no, there is no any problem because when I started giving him at that time he never knew what is kunu, when I gave him he will act as if he does not like it, I will now wait a little while before I will carry it and give him again. Like now the moment I give him he will just drink it. There is no problem with him taking the MNP

I: Thank you very much. You know there were some caregivers that complained that it was difficult for them to store the product with rodents eating it or others coming to take it. I would like to know your experience on such?

R: (cut in) is it not until you keep it where children will see it and take it, that one it is the fault of the caregivers. On that day when we were giving this MNP, they said even when the child drinks and remain, it should not be giving to any child and it should not be kept within the reach of children so that they will not go and carry it. From there we accepted it, and I took my own and kept it on top of my cupboard if not me, my husband, one lady and my mate nobody will reach there. Whenever I stand up to give him, the boy next to him will start crying that I should give him too and I will say no because they said it is only him that will take the product and nobody else should drink it.

I: Some caregivers again complained that it was difficult for them to store the product with rodents coming to eat it, what about you do you have similar experienced?

R: No no I will not lie to the rats, the way I promise to give my child this product that is how I have been given him. And again where I kept my product is not a place where rats will go and carry it. If I see the possibility of rat coming to take it I have a way of killing them after all I cover the product very well where by rats cannot even have access to it in the room

I: I sincerely appreciate the way you have been discussing with me but now I would like us to talk about any side effect that you think is the cause of this product?

R: Actually, I have not seen any side effect

I: There were some caregivers that said sometimes it could be difficult remembering to use the MNP, have you ever forgotten to give him in a day?

R: If I have forgotten or skip a day this medicine would have been more than this. The very day we were given this medicine towards afternoon, in the evening of that day, I make kunu use the product and gave my child, the next day I saw one of my colleague that we were giving the product together, I ask her have you stated giving your child the product, she said no that she was going to start today and I told her I started since yesterday in the evening. Up till today I don't use to forget and I have never skipped a day. There was a day my husband even asked me that have I be giving the medicine to the boy? I now said yes, I use to give him every day.

I: what advise can you give to those women that normally use to forget to give their children the MNP?

R: The advice I will give them since they said that rats do carried the products and they said that they do forget at times, it is not the issue of forgetfulness but they should be conscious of what they are doing and stick in one place by giving their children this product since the product is very helpful to the children. But the issue of rats coming to carry it, they should find a place where children will not reach since they said they do forget, the medicine should be kept inside the box of their clothes so that every morning they will go and open their box, remove one and give to their child but they should not rely on either they forget or rats came and carried it away. They should keep their mind in the medicine and be giving their children. That is the only way I feel I can advice them to do.

I: Thank you very much Ma. Previously you mentioned that you do mix the product with kunu and give to your child, could you please tell me what food you usually mix the product with?

R: Yes, on that day it was said even you can mix it with soup is that not so?

I: Yes

R: Up till today I have not try it with soup but I have been giving him with kunu and I have not try it with soup but they said you can use soup also.

I: Which type of kunu do you use?

R: It is akamu (pap) but in the first place I use grinding sorghum powdery grain cooked into a semi liquid form and gave him on that first day I was given the product. From that day I have been using pap even today I use pap

I: What time do you normally give him?

R: At times in the morning and at times again when the sun set but to be honest I usually give him in the morning like this time

I: At what time do you give him most often?

R: I mostly give him in the afternoon because in the morning he normally takes breast milk to his satisfaction, if I give him, he will refuse to take, and another reason why I normally give him in the morning it is because after taking breast milk in the morning I will allowed him to be seriously hungry. I will now use that opportunity and give him the MNP so that I can give him without him refusing to take it that is the reasons I always do like that to be honest with you.

I: Thank you very much; from your own experience do you think the baby notices that the kunu was mixed with something else whenever he takes it?

R: No, he has never noticed it; if you see him refusing to take it may be I have given him something to his satisfaction but outside that, he do take it like he is drinking water. And he will drink it all and I will still add for him the one without the MNP to drink

I: Previously you mentioned that his appetite has increased and he eats more now, could you please tell me what kind of food he eats more of now?

R: He has been eating food very well

I: What about breast milk has it increase or reduce the rate at which he takes breast milk?

R: If before he has been taking breast milk and even up till now he has not changed the way he takes breast milk

I: You said before he started taking the MNP he has not been taking kunu but now he is taking kunu

R: Yes

I: Now, apart from that kunu, is there any other foods he do take?

R: He eats, rice, kunu and everything that we cook that is available for him to eat

I: In other communities, the sharing of MNP was noticed, I would like for you to please tell me your experience about that?

R: Honestly, the way they told me not to give anybody even inside the house, and again you should not even fetch in the sense that it will remain, you should use the kunu that will be enough for him even if the children are around I should not give anybody. Is that not what they said should be done?

I: Yes it is like that, is there any of your friend you once remove some MNP and give to her to go and use for her child

R: No I have not done it, but I said to you that they said he is the only person I should give, and if I see that he is not taking it I should use spoon to be giving him to drink. And that I should not make the mistake of giving it to anybody even my friends or sisters he is the only person that will take his medicine for two months

I: Thank you very much, now if the child doesn't finish the kunu that is mixed with the MNP what do you do with the rest?

R: I told you I normally use ludayi (local spoon) to give him so there is no way it will remain and again after giving him I will add the one without the MNP for him to him to drink

I: You were given 60 sachets and told to use one per day, I want to know in your own opinion whether you think these 60 sachets would be enough for 60 days?

R: They should be giving one per day it will be enough

I: Why is it that one should be giving to him per day

R: It is because if you give him one he can be able to finish it, but if you give him more than one he might not finished and they said it is not good for the child to drink it and remain. And again I am scare of using two for him so that he drink and remain children may come and drink the remaining since they said nobody should take along with the child. If I give him that one in a day I am sure of him finishing it at once

I: If I understood your explanation, one per day is enough for the 60 days

R: Yes one per day is enough for the 60 days

I: Now, you have been calling this MNP medicine I want you tell me now do you considered it as food or medicine

R: Frankly speaking I will tell you two things: I really see it as a medicine and at the same time I am seeing it as food

I: Why do you said so

R: Because I really enjoyed it.

I: But if you are ask to choose one which one will you choose

R: I will choose that of the medicine

I: Why did you see it as food?

R: It is because when I started giving my child I really enjoyed it

I: Enjoyed it as how

R: When my child started taking the MNP he totally change as I have told you before his body changed

I: Which type of change are you really talking about?

R: I mean his body was looking good and he is eating well

I: Thank you very much. Am sure you were told this product is for free but what if you were asked to buy it, do you think you can buy it for some token amount of money?

R: Honestly I can buy it because I really enjoyed it

I: How much do you think you can buy it?

R: In my own opinion if they bring it for sale even if they say is five hundred naira (N500) I will buy

I: Actually I thank you for rich information. Could you please tell me some ways you think would be effective for promoting the MNP in this community so that they would comply with using it appropriately?

R: If the people are close to me I will tell them that the medicine is good because I have used it. And again the message can be pass through the district head to our husbands and it will reach us the caregivers

I: Who are the people must influence your opinions and knowledge about feeding practices?

R: It is my husband I always ask for any feeding practice, I will tell that the child has a stage whereby we can start giving him kunu

I: I mean who are the people that always give you advice to give your child this or that

R: Honestly, if it is that one it is only my husband, I and my mother in-law

I: Ok, thank you very much but I would like to also know who influenced your decision to give the MNP to your child.

R: It is the elder brother of my husband that came and call me, when he called me together with one man, he explained to me that this medicine should be giving to the child and the man now ask me some questions and I answered him. They now said it is the medicine they will give us;

they now give us and told us what to do with the medicine. I now left and continue to do what they ask me to do as in every morning I will use one sachet for my child and my husband and my mother in-law will be reminding me not to forget to give the medicine to the child

I: Where would be a good place to disseminate health information related to MNP?

R: It is the secretary house

I: Which secretary

R: Secretary Rafin Gora the elder brother of my husband

I: So the elder brother of your husband is the secretary of Rafin Gora. What forms of media do you prefer for receiving MNP messages?

R: Let it be through the radio

I: Do you mean all the members of the community have radio?

R: Until you find out first

I: What about in the hospital

R: Honestly I will tell you the truth, we are in raining now and if it is not raining season some people are stubborn, because of that if I said in the hospital, some people if not sickness nothing will carry them to the hospital, if you see somebody in the hospital that means he is sick. The hospital is located in the bush not within the village; it will be hard for people to be seeing in the hospital like that. But if they can bring it to the people, they will accept it. That is the only thing I can say

I: But do you think poster can help to show people how they can feed the child?

R: Yes you can make poster it will go a long way in helping people

I: Thanks so much for that suggestions. Tell me about specific examples of other successful health education campaigns in this community.

R: (long silent) as for me I really enjoyed the medicine because of that if the medicine is brought here, I will still go and collect and I will continue to advice others not to think twice in collecting it

I: You said earlier that the medicine should be collected from the secretary, why did you said so

R: It is because I trust him

I: As you trust him do you think the rest of the community members also trust him

R: Yes, they also trusted him

I: Why did you say that the community members have trust on the secretary?

R: It is because if there is anything in this community, he is the one that do follow people and make announcement and the people will gather round him

I: That's so good, ok let's say you want to explain the product to your sister on how to use it, what would you tell her?

R: What I will tell her is that there is a medicine that has been brought to us and we have tested it on our children and it is effective because my child was not eating well but now he can eat very well. After that, I normally mix it with kunu for him to drink and even now he eats tuwo and that the medicine is good and she should not hink otherwise about the medicine. If she is eventually given the medicine she should be very serious in giving her child. She should test it and see for herself because I have already been convinced.

I: If you will advice to administer the MNP to the child what will you tell them?

R: What I will tell them based on this MNP is that the medicine we were giving they should not give to the child in the sense that it will remain, and again they should not give the medicine with dirty hands or dirty cup it should be giving with in a clean environment and with a clean cup. If the person finger is dirty, she should use spoon to stir the medicine. And she should not allow any child or children to share with the child and she should not share it with the neighbours around her.

I: I sincerely appreciate you a lot, now we have come to the end of this discussion but before I go is there anything you would like to share with me about your experience since you started using this product?

R: I have a question and my question is that the way this medicine was giving to only few of us women are they going to bring more to carter for the rest of the community members?

I: Do yu have any particular question on the MNP?

R: The question I will ask is that if the child grows up and the medicine still remains can the medicine still be giving to him?

I: If he grows as how

R: Is like you said they will still bring more

I: Yes but is for children between the age of 6-23 months

R: Okay

I: Did you understand now

R: Yes

I: Do you have any advice on this MNP on how to share it in the community?

R: The only advice I will give is that when you bring the medicine to the secretary, anybody that has a child of that range should be giving the medicine to use. They should bring enough for everybody in the community so that they too can testify the effectiveness of it based on what I have told them

I: Thank you very much, that is all.
